# The complete mitochondrial genome of the Egyptian honey bee, *Apis mellifera lamarckii* (Insecta: Hymenoptera: Apidae)

**DOI:** 10.1080/23802359.2017.1325343

**Published:** 2017-05-12

**Authors:** Amin Eimanifar, Rebecca T. Kimball, Edward L. Braun, Dahi M. Moustafa, Nizar Haddad, Stefan Fuchs, Bernd Grünewald, James D. Ellis

**Affiliations:** a Honey Bee Research and Extension Laboratory, Entomology and Nematology Department, University of Florida, Gainesville, FL, USA;; b Department of Biology, University of Florida, Gainesville, FL, USA;; c Department of Agricultural Technical Education Directorate, Ministry of Education Directorate of Education in Minia Governorate, Minia Governorate, Egypt;; d Bee Research Department, National Center for Agricultural Research and Extension, Amman, Jordan;; e Institut für Bienenkunde, Polytechnische Gesellschaft, Goethe-Universität Frankfurt am Main, FB Biowissenschaften, Oberursel, Germany

**Keywords:** Egyptian honey bee, mitogenome, next-generation sequencing, Apis mellifera lamarckii

## Abstract

The complete mitochondrial genome of the western honey bee subspecies *Apis mellifera lamarckii* was sequenced. This mitochondrial genome is 16,589 bp in length with 37 classical eukaryotic mitochondrial genes and an A + T-rich region. Gene directions and arrangements are similar to those of other *Apis* mitogenomes. Seven genes begin with ATT, four with ATG, and two with ATA (none with ATC) and all genes terminate with TAA. Four genes are encoded on the heavy strand and nine are encoded on light strand. All of the 22 tRNA genes, ranging from 66 to 80 bp, have a typical cloverleaf structure. A phylogenetic tree showed that *A.m. lamarckii* clusters with other *A. mellifera* subspecies, as expected.

*Apis mellifera lamarckii* Cockerell, 1906 is a western honey bee subspecies naturally distributed in Egypt and the Sudan along the Nile Valley. Its behaviour is similar to *A.m. intermissa* (Fasasi et al. 2011; Hu et al. 2016). It is well adapted to the local conditions and pests of the region. However, *A.m. lamarckii* is considered to exhibit high levels of defensive behaviour and to be an inferior honey producer by some beekeepers (Soliman Kamel & Sheppard 2003). Sequencing of mitogenomes from unstudied subspecies will promote our understanding of the mitogenome evolution and diversity in *Apis*. For this study, an adult honey bee worker of *A.m. lamarckii* was obtained from the Ruttner Bee Collection at the Bee Research Institute at Oberursel, Germany (Voucher No. 1842, W.S. Sheppard, Egypt, 1990, 27°14N, 31°07E) and its mitogenome was reported (GenBank accession No. KY464958). The identity of the bee was confirmed morphometrically by the Institute staff. Extraction and quantification of genomic DNA were performed according to the methods described by Eimanifar et al. (2016).

A genomic library was constructed from genomic DNA using a Kapa Hyper Prep Kit (Kapa Biosystems, Woburn, MA) with a paired-end read (2 × 150) followed by sequencing on Illumina Hi-Seq 3000/4000 (San Diego, CA). The sequencing reads were trimmed using Trimmomatic v0.35 (Bolger et al. 2014) and mapped to the reference honey bee mitochondria (*A.m. ligustica*, L06178.1, the Italian honey bee) using bowtie v2.2.9 (Langmead & Salzberg 2012). The subset of reads was then mapped to the reference using breseq v 0.28.1 (Deatherage & Barrick 2014) to verify that the coverage was correct and uniform. The resulting reads were adjusted and assembled using Spades v3.9.0 (Bankevich et al. 2012). The resulting contigs were blasted against the reference sequence using NCBI blast package v2.2.19 and the best contigs were identified. These contigs were then used as the reference for breseq mapping as a final step to verify that the coverage was even and met expectations. The assembled mitogenome was aligned with those of other *Apis* species and subspecies using Mesquite v 3.10 (Maddison & Maddison 2016) and were adjusted manually.

The complete sequence of *A.m. lamarckii* was 16,589 bp in length with 13 protein-coding genes (PCGs), 22 transfer RNAs (tRNA), 2 ribosomal RNAs (rRNA), and 1 putative control region (CR). The overall base composition of the *A.m. lamarckii* mitogenome was A (43.3%), T (41.6%), C (9.5%), and G (5.6%), respectively. The gene content, structure, and arrangement are similar to those observed in other *Apis* mitogenomes (Eimanifar et al. 2016). Four mitochondrial genes are encoded on the H-strand and nine on the L-strand. The ATP6 and ATP8 overlap, as they do in other *Apis* mitogenomes. The most common start codon was ATT (in seven PCGs); others began with ATG (four PCGs) and ATA (two PCGs). All PCGs terminated with a TAA stop codon.

The 16S rRNA and 12S rRNA were 1340 and 785 bp long respectively, with an average of 84.2% and 81.4% AT, respectively. The 22 tRNA genes ranged from 66 to 80 bp in size. All tRNAs were folded into a typical cloverleaf-shaped secondary structure as identified by tRNAscan-SE (Lowe & Eddy 1997). The CR was 843 bp long and 95.6% AT.

The phylogenetic position of *A.m. lamarckii* was determined by maximum likelihood using concatenated nucleotide sequence of the 13 PCGs and 2 rRNAs genes using RAxML 8.2.0 on the CIPRES science gateway (Miller et al. 2010). The tree was rooted with *A.m. ligustica* with 1000 bootstrap replicates*. Apis mellifera lamarckii* was well supported as belonging to *A. mellifera* (Figure 1). The *p*-distance between *A.m. lamarckii* and *A.m. mellifera* ranged from 0.0047 to 0.0132, and between *A.m. lamarckii* and *A.m. intermissa* was 0.0117.

**Figure 1.**
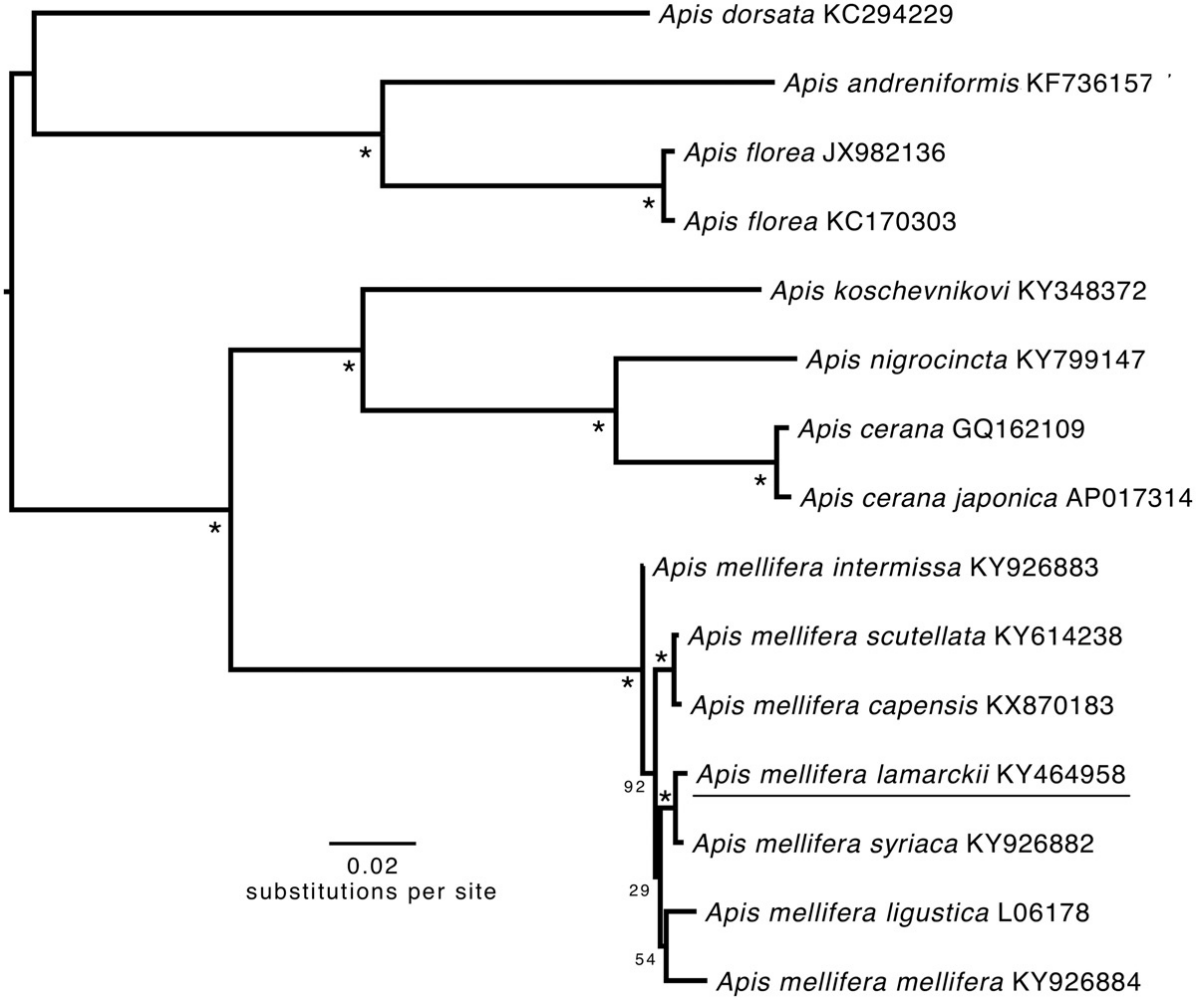
Phylogenetic tree constructed with *A.m. lamarckii* and 14 other *Apis* species and subspecies mitogenomes. It was constructed based on the alignment of the concatenated dataset of 13 PCG and 2 rRNA genes using the maximum-likelihood method within the RAxML package. The bootstrap support values are generated using 1000 replications. The bootstrap values are indicated behind each node. GenBank sequences are listed, followed by species names.

We believe that this study provides information on the genetic resources of *Apis* subspecies and could be helpful to elucidate the evolutionary relationships and species-specific markers in *Apis* based on the complete mitochondrial genome data, especially that the *A.m. lamarckii* threatened by despairing or genetically diluted due cross breeding with imported commercial queens.
